# Functional Disability in the Context of Cancer Diagnosis: Examining the Within- and Between-Person Associations

**DOI:** 10.1093/geroni/igaa057.1464

**Published:** 2020-12-16

**Authors:** Rumei Yang, Yin Liu, Elizabeth Fauth

**Affiliations:** 1 Nanjing Medical University, Nanjing, China; 2 Utah State University, Logan, Utah, United States

## Abstract

Physical functioning is essential for independent living and aging in place among older adults. Although older adults show a general trend of increasing functional disability, it was unclear how cancer diagnosis, which also has an increased incidence rate among older adults, may potentially aggravate the progress of functional decline at the within- and between-person levels. Using 3 waves of the China Health and Retirement Longitudinal Study (CHARLS, 2011-2015), the current study examined the trajectory of functional disability and associated factors over 5 years among older adults with (n = 139) and without cancer (n = 212), who were matched on key demographic characteristics. We considered four contributing factors to disability as specified in the Disablement Process model as covariates, in the context of cancer diagnosis timing at both the within- and between-person levels. These factors were psychological (i.e., depressive symptoms), physical limitations (i.e., pain, fall, and frailty), cognitive impairment (i.e., self-reported memory problems and word recall), and environmental constraints (i.e., social contact and availability of support). Consistent with the literature, there was a general trend of increasing functional disability among all participants over time. At the within-person level, although cancer diagnosis timing was associated with higher levels of functional difficulty, we found a slower increasing rate over time. At the between-person level, functional disability trajectory was not associated with cancer diagnosis timing. These findings suggest that maintenance of physical functioning should be considered at the within-person level, and personalized care is recommended for Chinese older adults with cancer.

